# Stratified analysis and clinical significance of elevated serum triglyceride levels in early acute pancreatitis: a retrospective study

**DOI:** 10.1186/s12944-017-0517-3

**Published:** 2017-06-27

**Authors:** Jianhua Wan, Wenhua He, Yin Zhu, Yong Zhu, Hao Zeng, Pi Liu, Liang Xia, Nonghua Lu

**Affiliations:** Department of Gastroenterology, The First Affiliated Hospital of Nanchang University, 17 Yongwaizheng Street, Nanchang, Jiangxi 330006 People’s Republic of China

**Keywords:** Acute pancreatitis, Hypertriglyceridemia, Triglyceride

## Abstract

**Background:**

Hypertriglyceridemia is one of the three most common causes of AP, which is associated with the AP prognosis that has not been clearly defined.

**Methods:**

In this retrospective study, 1539 AP patients, who had serum triglyceride (TG) levels measured within the first 72 h, were assessed. The study groups consisted of patients with normal, mild, moderate, and severe/very severe HTG levels based on the Endocrine Society Clinical Practice Guidelines. We collected baseline demographic information, laboratory values, complications, and clinical outcome data in different HTG severity groups to analyze the clinical significance of elevated TG levels in AP.

**Results:**

Our study included 1539 AP patients; of these, 1078 (70%) had a normal TG levels, and 461 (30%) had elevated TG levels. The rates of severe AP increased in HTG groups of increasing severity (4% vs. 8% vs. 12%; *P*_trend_ < 0.001). acute necrotic collection (ANC) and pancreatic necrosis developed in 32 and 39 of 112 patients (29% and 35%) (*P*_trend_ = 0.001; *P*_trend_ = 0.001) in the severe/very severe HTG group, respectively. The proportion of persistent organ failure (POF), multiple organ failure (MOF), and persistent Systemic Inflammatory Response Syndrome (SIRS) increased with higher grades of HTG (*P*_trend_ < 0.001; *P*_trend_ < 0.001; *P*_trend_ < 0.001). The ICU admission rate was higher in the severe/very severe HTG group (57/112 patients; 51%; *P*_trend_ < 0.001). A logistic multivariate regression analysis showed a positive correlation between HTG and certain AP complications.

**Conclusion:**

In addition to other factors, an elevated TG level could be associated with the severity and prognosis of AP, including pancreatic necrosis, POF, MOF, persistent SIRS, ICU admission, and mortality.

## Background

Acute pancreatitis (AP) is a complex inflammatory disease that locally involves the pancreas as well as systemic organs. The AP incidence is approximately 70 cases per 100,000 individuals worldwide [1]. Mild acute pancreatitis (MAP) often has a good prognosis; however, 15–20% of AP patients develop severe acute pancreatitis (SAP), which has higher morbidity and mortality rates. Clinical findings associated with a severe course in the initial risk assessment include patient age, body mass index, the presence of Systemic Inflammatory Response Syndrome (SIRS), signs of hypovolemia, such as an elevated blood urea nitrogen (BUN) and hematocrit (HCT), the presence of pleural effusions and/or infiltrates, altered mental status, and other factors [1].

Hypertriglyceridemia (HTG), which is one of the three most common causes of AP, occurs in 14.3% of AP patients [2]. Compared to other etiologies, patients with hyperlipidemia acute pancreatitis (HLAP) show a higher mortality rate, more severe prognoses and more frequent local complications [3]. However, some studies have reported no differences in the prognoses between these conditions [4]. It is well known that the serum triglyceride (TG) level is an indicator of an HLAP diagnosis. Many reports have shown that TG elevation on admission for AP is a predictor of a poor prognosis as well as local and systemic complications. These conflicting findings were limited to studies with small sample sizes. The effect of elevated TG levels on the AP prognosis has not been clearly defined. In this retrospective study, we aimed to clarify the effect of elevated TG levels in AP and its prognosis by analyzing a large sample dataset.

## Methods

This is a retrospective single-center observational study. The study period was 8 years, occurring between January 1, 2005 and December 31, 2013. The study was approved by the ethics committee of The First Affiliated Hospital of Nanchang University (No. 2011001).

### Patient selection

**Fig. 1:**
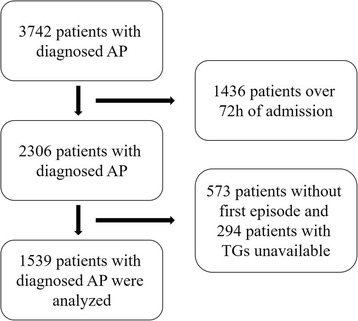
The selection process for patients in a flow chart. AP, acute pancreatitis

The data were collected from an electronic medical database. Patients who were admitted to our hospital with an AP diagnosis and had serum TG levels measured within 72 h of presentation were included in the study. The selection process for patients is shown in a flow chart (Fig. 1). AP was diagnosed according to the Chinese guidelines for the diagnosis and treatment of AP [5], which include the presence of at least two of the following three criteria: (1) classic abdominal pain; (2) elevation of serum amylase and/or lipase to three times the upper limit of normal; and (3) radiographic evidence of AP. The classification of SAP and moderately severe AP (MSAP) was established using the 2012 revision of the Atlanta classification and definitions by the international consensus [6].

### HTG definition and classification

The diagnosis and severity classification of HTG were based on the Endocrine Society Clinical Practice Guidelines [7]. An HTG diagnosis was defined as serum TG levels ≥1.7 mmol/L. HTG was also classified into different severity categories: mild (serum TG levels of 1.7–2.3 mmol/L), moderate (2.3–11.2 mmol/L), severe (11.2–22.4 mmol/L), and very severe (≥22.4 mmol/L). All values were measured within the first 24 h of admission.

### Data collection

Baseline demographic data, including age, gender, medical history, admission number, and date, were collected for all patients in the study. We collected data on the patients’ vital signs at admission, hemogram, blood biochemical parameters, and blood gas analysis.

### Statistical methods

Statistical analyses were performed using IBM SPSS software, version 20.0 (Statistical Package for Social Sciences, Chicago, USA). Numerical data are expressed as the means ± SD, and categorical variables are expressed as the means (ratios). For continuous variable comparisons, an independent sample Student’s t test was used for two groups, and Cuzick’s trend test was used for multiple groups. Categorical variables were evaluated using the χ^2^ and Fisher’s exact tests. Logistic multivariate regression analyses were used for risk factors with categorical variables. A value of *P* < 0.05 was considered statistically significant.

## Results

### Comparison of the general patient information

The study included 1539 AP patients; of these, 1078 (70%) had normal TG levels (0.80 ± 0.37 mmol/L), and 461 (30%) had elevated TG levels (8.42 ± 8.91 mmol/L). The 461 patients with HTG were more commonly male (70% vs. 49%; *P* < 0.001) and younger (median age in years 44 vs. 55; *P* < 0.001) compared to patients with normal TG levels. A higher proportion of acute biliary pancreatitis was observed in the normal TG group, and more cases of alcoholic and HTG pancreatitis were found in the HTG group. Histories of diabetes mellitus (3% vs. 13%; *P* < 0.001), smoking (20% vs. 36%; *P* < 0.001), and alcoholism (17% vs. 37%; *P* < 0.001) were more common in the HTG group. No significant difference was identified with regard to hypertension (19% vs. 18%; *P* = 0.559). The baseline pancreatitis characteristics and etiologies in both groups are shown in Table 1.

**Table 1:** Population baseline characteristics between AP patients without vs. with HTG

	Normal TGs	HTG	
Variable	*N* = 1078	*N* = 461	*P*
Median age, years (IQR)	56 (44–67)	42 (35–51)	<0.001
Sex, N (%)			<0.001
Male	525 (49)	323 (70)	
Female	553 (51)	138 (30)	
Pancreatitis etiology, N (%)			<0.001
Biliary	800 (74)	108 (23)	
Alcoholism	54 (5)	48 (10)	
Hypertriglyceridemia	0 (0)	120 (26)	
Idiopathic	133 (12)	29 (6)	
Others	91 (8)	156 (34)	
Comorbidities, N (%)			
Hypertension	203 (19)	81 (18)	0.559
Diabetes mellitus	36 (3)	62 (13)	<0.001
Smoker	212 (20)	167 (36)	<0.001
Alcoholism	184 (17)	172 (37)	<0.001

*TG* triglyceride, *HTG* hypertriglyceridemia, *N* number, *IQR* Inter Quartile Range

The stratified analysis of the HTG group showed that patients with greater HTG severity were more likely to be younger (*P*_trend_ < 0.001), male (*P*_trend_ < 0.001), have diabetes mellitus (*P*_trend_ < 0.001), be alcoholic (*P*_trend_ < 0.001), and smoke (*P*_trend_ < 0.001), as presented in Table 2. The biliary etiology was predominant among patients with normal TG levels (74%), whereas 96% of patients with severe/very severe HTG had HTG-induced AP (Table 2).

**Table 2:** Population baseline characteristics between hypertriglyceridemia categories

	Serum TG Level (mg/dL) within 72 h of hospital admission				
	Normal	Mild	Moderate	Severe and very severe	
Variable	*N* = 1078	*N* = 107	*N* = 242	*N* = 112	*P*_trend_
Median age, years (IQR)	56 (44–67)	49 (38–57)	42 (35–50)	39 (32–46)	<0.001
Sex, N (%)					<0.001
Male	525 (49)	60 (56)	173 (71)	90 (80)	
Female	553 (51)	47 (44)	69 (29)	22 (20)	
Etiology, N (%)					<0.001
Biliary	800 (74)	52 (49)	55 (23)	1 (1)	
Alcoholism	54 (5)	18 (17)	27 (11)	3 (3)	
Hypertriglyceridemia	0 (0)	0 (0)	12 (5)	108 (96)	
Idiopathic	133 (12)	11 (10)	18 (7)	0 (0)	
Others	91 (8)	26 (24)	130 (54)	0 (0)	
Comorbidities, N (%)					
Hypertension	203 (19)	27 (25)	33 (14)	21 (19)	0.068
Diabetes mellitus	36 (3)	13 (12)	29 (12)	20 (18)	<0.001
Smoker	212 (20)	28 (26)	90 (37)	49 (44)	<0.001
Alcoholism	184 (17)	33 (31)	92 (38)	47 (42)	<0.001

*TG* triglyceride *N* number, *IQR* Inter Quartile Range

Laboratory parameters on admission were compared among patients with different HTG severity categories. Some parameters, such as HCT, lactate dehydrogenase (LDH), total cholesterol (TC), and blood glucose (GLU), increased with higher grades of HTG (*P*_trend_ < 0.001). Other parameters recorded are presented in Table 3 **.**

**Table 3:** Laboratory test values on admission between hypertriglyceridemia categories

	Serum TG Level (mg/dL) within 72 h of hospital admission				
	Normal	Mild	Moderate	Severe and very severe	
Variable	*N* = 1078	*N* = 107	*N* = 242	*N* = 112	*P*_trend_
WBC, ×10^3^/μL	12.65 ± 5.18	13.45 ± 4.96	12.97 ± 5.07	13.80 ± 4.25	0.066
NEU, ×10^3^/μL	85.77 ± 8.60	85.24 ± 6.99	85.30 ± 8.02	84.54 ± 10.14	0.454
Hb, g/L	132 ± 22	135 ± 29	142 ± 24	155 ± 29	<0.001
HCT, %	38.94 ± 5.69	40.23 ± 7.54	40.44 ± 7.24	42.94 ± 6.54	<0.001
PLT, ×10^3^/μL	170 ± 67	167 ± 56	170 ± 68	185 ± 71	0.128
ALT, U/L	123 ± 130	115 ± 152	60 ± 64	45 ± 54	<0.001
AST, U/L	113 ± 146	134 ± 230	70 ± 129	53 ± 76	<0.001
TBIL, U/L	32.65 ± 30.67	33.22 ± 34.75	27.43 ± 23.80	20.59 ± 16.45	<0.001
LDH, U/L	318 ± 133	362 ± 226	364 ± 242	407 ± 272	<0.001
CK, U/L	127 ± 198	196 ± 538	197 ± 459	195 ± 255	<0.001
TG, mmol/L	0.80 ± 0.37	1.98 ± 0.17	5.02 ± 2.45	21.92 ± 8.18	<0.001
TC, mmol/L	4.41 ± 0.83	4.73 ± 1.04	5.11 ± 1.26	8.43 ± 4.49	<0.001
AMY, U/L	864 ± 828	599 ± 744	470 ± 527	525 ± 472	<0.001
GLU, mmol/L	7.31 ± 3.10	8.74 ± 4.23	9.05 ± 5.00	13.20 ± 6.75	<0.001
Ca, mmol/L	2.12 ± 0.25	2.05 ± 0.27	2.08 ± 0.30	2.02 ± 0.32	<0.001
BE	−2.00 ± 2.93	−2.48 ± 3.68	−2.92 ± 3.72	−5.65 ± 5.43	<0.001

*TG* triglyceride, *N* number, *WBC* white blood count, *NEU* neutrophile granulocyte, *Hb* hemoglobin *HCT* hematocrit, *PLT* Platelets, *ALT* alanine transaminase, *AST* aspartate aminotransferase, *TBIL* total bilirubin, *LDH* lactate dehydrogenase, *CK* creatine kinase, *TC* total cholesterol, *AMY* amylase, *GLU* blood glucose, *BE* base excess

### Clinical outcomes

The 1539 AP patients included 327 (21%) acute peripancreatic fluid collection (APFC) cases, 242 (16%) acute necrotic collection (ANC) cases, 336 (22%) pancreatic necrosis cases, 345 (22%) persistent organ failure (POF) cases, 101 (7%) multiple organ failure (MOF) cases, 373 (24%) persistent SIRS cases, 438 (28%) ICU admissions, 87 patients (6%) who succumbed to their illness, and 37 (2%) high volume hemofiltration (HVHF) cases. The median hospital length of stay was 9 days.

The rates of severe AP increased as the HTG severity classification of patients increased (4% vs. 8% vs. 12%; *P*_trend_ < 0.001). ANC and pancreatic necrosis developed in 32 and 39 of 112 patients (29% and 35%) (*P*_trend_ = 0.001; *P*_trend_ = 0.001) with severe/very severe HTG, respectively. The proportion of POF, MOF and persistent SIRS increased with higher grades of HTG (*P*_trend_ < 0.001; *P*_trend_ < 0.001; *P*_trend_ < 0.001). The need for ICU admission was higher in the severe/very severe HTG group, accounting for 57/112 patients (51%; *P*_trend_ < 0.001). One patient with a normal TG level required HVHF (0.1%), and 29 patients with severe/very severe HTG required HVHF (26%) (*P*_trend_ < 0.001). No significant difference was noted in the development of APFC, length of stay, and mortality among the groups as shown in Table 4.

**Table 4:** Incidence of complications between hypertriglyceridemia categories

	Serum TG Level (mg/dL) within 72 h of hospital admission				
	Normal	Mild	Moderate	Severe and very severe	
Variable	*N* = 1078	*N* = 107	*N* = 242	*N* = 112	*P*_trend_
Severity classification, N (%)					<0.001
MAP	424 (39)	43 (40)	85 (35)	21 (19)	
MSAP	440 (41)	34 (32)	98 (41)	49 (44)	
SAP	214 (20)	30 (28)	59 (24)	42 (38)	
APFC, N (%)	221 (21)	18 (17)	59 (24)	29 (26)	0.125
ANC, N (%)	150 (14)	17 (16)	43 (18)	32 (29)	0.001
Pancreatic necrosis, N (%)	210 (20)	25 (23)	62 (26)	39 (35)	0.001
OF, N (%)	368 (34)	48 (45)	91 (38)	59 (53)	<0.001
POF, N (%)	214 (20)	30 (28)	59 (24)	42 (38)	<0.001
MOF, N (%)	31 (3)	9 (8)	23 (10)	12 (11)	<0.001
Persistent SIRS, N (%)	221 (21)	28 (26)	70 (29)	54 (48)	<0.001
Median hospital days (IQR)	9 (6–14)	9 (5–13)	10 (6–14)	12 (7–17)	0.079
Median ICU days, (IQR)	0	0 (0–1)	0 (0–2)	1 (0–7)	0.045
Admission to ICU, N (%)	267 (25)	31 (29)	83 (34)	57 (51)	<0.001
Mortality, N (%)	53 (5)	11 (10)	15 (6)	8 (7)	0.112
HVHF, N (%)	1 (0.1)	1 (1)	6 (3)	29 (26)	<0.001

*TG*, triglyceride, *N* number, *MAP* mild acute pancreatitis, *MSAP* moderately severe acute pancreatitis, *SAP* severe acute pancreatitis, *APFC* acute peripancreatic fluid collection, ANC acute necrotic collection, *OF* organ failure, *POF* persistent organ failure, *MOF* multiple organ failure, *IQR* Inter Quartile Range, *HVHF* high volume hemofiltration

AP patients with HTG pancreatitis had a higher proportion of MSAP (44%) and SAP (34%). TG and GLU levels increased as the AP severity increased (*P*_trend_ < 0.001; *P*_trend_ < 0.001). A higher proportion of AP patients succumbed to their illness in the SAP group than in the MSAP and MAP groups (16% vs. 4% vs. 1%; *P*_trend_ < 0.001). Similarly, 25 cases (7%) of HVHF were observed in the SAP group, which was at a rate higher than the rates observed in the MSAP (2%) and MAP (0.2%) groups (*P*_trend_ < 0.001) as shown in Table 5.

**Table 5:** Comparison of baseline clinical characteristics and outcomes between AP patients on severity classification

	MAP	MSAP	SAP	
Variable	*N* = 573	*N* = 621	*N* = 345	*P*_trend_
Etiology, N (%)				0.004
Biliary	354 (39)	358 (39)	196 (22)	
Alcoholism	34 (33)	44 (43)	24 (24)	
Hypertriglyceridemia	26 (22)	53 (44)	41 (34)	
Idiopathic	68 (42)	69 (43)	25 (15)	
Others	91 (37)	97 (39)	59 (24)	
TG, mmol/L	2.30 ± 4.65	3.10 ± 5.97	4.34 ± 7.64	<0.001
GLU, mmol/L	6.98 ± 2.87	8.26 ± 4.38	9.74 ± 5.09	<0.001
Admission to ICU, N (%)	27 (5)	196 (32)	215 (62)	<0.001
Persistent SIRS, N (%)	47 (8)	141 (23)	185 (54)	<0.001
Mortality, N (%)	7 (1)	26 (4)	54 (16)	<0.001
Pancreatic necrosis, N (%)	0	181 (29)	155 (45)	<0.001
HVHF, N (%)	1 (0.2)	11 (2)	25 (7)	<0.001

*MAP* mild acute pancreatitis *MSAP* moderately severe acute pancreatitis, *SAP* severe acute pancreatitis, *N* number, *TG* triglyceride, *GLU* blood glucose, *HVHF* high volume hemofiltration

A logistic multivariate regression analysis showed an association between TG levels and complications after adjusting for age ≥ 60 years, sex, pancreatitis etiology, hypertension, diabetes mellitus, smoking, and alcoholism. A higher TG level was independently associated with pancreatitis prognosis, including ANC, pancreatic necrosis, OF, POF, MOF, persistent SIRS, ICU admission, and mortality, as shown in Table 6.

**Table 6:** Multivariate analysis showing association of triglyceride level with complications after adjusting for age, sex, pancreatitis etiology, hypertension, diabetes mellitus, smoker, alcoholism

Complication	B	OR	*P*
APFC	0.13	1.13	0.074
ANC	0.27	1.31	<0.001
Pancreatic necrosis	0.19	1.20	0.007
OF	0.29	1.34	<0.001
POF	0.28	1.32	<0.001
MOF	0.48	1.61	<0.001
Persistent SIRS	0.31	1.36	<0.001
Admission to ICU	0.39	1.48	<0.001
Mortality	0.25	1.29	0.041

*APFC* acute peripancreatic fluid collection, *ANC* acute necrotic collection, *OF* organ failure, *POF* persistent organ failure, *MOF* multiple organ failure

## Discussion

We conducted a single-center retrospective study to analyze serum TG levels during early AP. Regardless of the underlying etiology of pancreatitis, this study provides a larger sample size and more reliable evidence than other previous studies [3, 4, 8, 9]. Above all, our study shows that HTG may be associated with more prognostic indicators, such as pancreatic necrosis, persistent SIRS, and the need for admission to the ICU.

Previous research has shown that obesity is a definite risk factor for morbidity and in-hospital mortality from AP and may serve as a prognostic indicator [10]. In a basic research study, Patel et al. noted that in obese mice, mild cerulein AP develops into SAP with greater cytokine and unsaturated fatty acid (UFA) levels and increased MOF [11]. UFAs are generated via lipolysis of visceral fat by pancreatic lipases, which cause mild AP to develop into SAP independently of pancreatic necrosis and the inflammatory response [11]. Eighty to 90 % of the adipocyte volume is composed of TGs, which can be hydrolyzed by lipases that are released during pancreatitis; serum TGs and adipose tissue are hydrolyzed, generating free fatty acids (FFAs) [12, 13]. Elevated TGs and FFAs are high risk factors that lead to toxic effects and are necessary to evoke damage to isolated pancreatic acinar cells [14]. Hyperlipidemia intensifies cerulein-induced AP associated with activation of protein kinase C in rats [15]. Another study reported that when AP induced by non-HTG causes is complicated with TG elevation, the AP disease course shows a trend for aggravation [3].

SIRS that persists for 48 h or more after symptom onset is indicative of a poor prognosis [16]. In our study, the proportion of persistent SIRS in severe/very severe HTG patients was 48%, which was much higher than that in the other groups (*P* < 0.001). Early reports have indicated the respiratory insufficiency that is seen in AP could be mediated by TG elevations affecting pulmonary gas exchange and mechanics [17]. In the early stage, the ANC incidence in AP patients with elevated TGs was significantly higher than that in patients without elevated TGs. Additionally, AP patients with higher HTG levels also showed more severe pancreatic necrosis after four weeks. However, a prospective cohort study did not show any significant association between HTG and pancreatic necrosis regardless of the underlying AP etiology [8]. One limitation of cohort studies was that the sample sizes were smaller than that of our study. Previous reports have also shown a higher prevalence of pancreatic necrosis in patients with HTG-induced AP [3, 18, 19]. However, the incidence of APFC during the early stage that was associated with elevated TG levels was not significantly different among AP patients.

Recently, several studies have reported an effect of HTG on the clinical course of AP. Charlesworth et al. reported that HTG-induced AP was present in 2.3% of patients presenting with AP [20]. Pedersen et al. reported that non-fasting mild-to-moderate HTG levels of 177 mg/dL (2 mmol/L) and above were associated with a high risk of AP in 116,550 individuals [21]. Tariq et al. [22] showed that a TG level of ≥2.26 mmol/L on admission of AP patients was an independent predictor of developing local and systemic complications, the hospital length of stay, admission to the ICU, and ICU length of stay. In our study, a higher proportion of AP patients with high TG levels were admitted to the ICU and for longer periods of time. However, other studies have shown that an increased TG level may only represent a symptom associated with AP, as no significant relationship was found between TG levels and the severity and prognosis of AP patients [23].

The determinant of the severity of AP during the early phase is primarily the presence and duration of organ failure [6]. The revised Atlanta classification system defines POF as SAP, and the presence of transient organ failure or local or systemic complications is defined as MSAP [6]. The proportion of POF and SAP in patients with elevated TGs increases gradually as the TG level is elevated. Similarly, the proportion of MOF was 10% in the moderate HTG group and 11% in the severe and very severe HTG group, which was higher than the in normal TG group (3%). Logistic multivariate regression analysis also supported the hypothesis that an elevated TG level could be associated with AP severity and prognosis, as well as pancreatic necrosis, POF, MOF, persistent SIRS, ICU admission, and mortality. Our study had several limitations. First, this study was retrospective, and thus, a larger prospective study is needed. Second, we only selected AP patients within 72 h of disease onset. Third, we did not have TG records for all patients in our hospital database, and therefore, an unintended bias may have occurred.

## Conclusion

In summary, a more severe form of pancreatitis was observed in the higher TG groups than that observed in the lower TG groups, which implies that an elevated TG level may be associated with a poor prognosis, as indicated by a discrete proportional trend in our AP patients.
